# Empowering pharmacists in Canada’s drug shortage response

**DOI:** 10.1177/17151635251365666

**Published:** 2025-10-04

**Authors:** Peter Chengming Zhang, Mina Tadrous

**Affiliations:** Leslie Dan Faculty of Pharmacy, University of Toronto, Toronto, ON; Southlake Health, Newmarket, ON; Leslie Dan Faculty of Pharmacy, University of Toronto, Toronto, ON; Women’s College Research Institute, Toronto, ON (Tadrous)

## Introduction

Drug shortages in Canada have become an escalating concern within the health care system. In 2022 alone, there were 3098 reported drug shortages, a sharp increase of 15% compared to the previous year.^1^ These drug shortages reflect not only the impacts of global supply chain fragility and geopolitical instability but also the limitations of Canada’s domestic response infrastructure.

While policy-makers and public health authorities grapple with long-term prevention and crisis management solutions, pharmacists are increasingly the first line of defense, tasked with managing disruption in real time while constrained by outdated or inconsistent regulations. In most provinces, pharmacists are not empowered to adequately respond to drug shortages. This dynamic not only disrupts patient access to necessary medications but also creates more workload for prescribers and pharmacists alike.

## Pharmacist adaptations as a response to drug shortages

The impact of drug shortages is broad and can vary widely.^2^ For example, during the 2018 recall of valsartan, a commonly prescribed angiotensin II receptor blocker, approximately 160,000 patients in Canada were likely forced to switch to alternative therapies within 1 month.^3^ These transitions, though clinically necessary, often strain the health care system with additional physician appointments, new prescriptions, repeat blood pressure testing, and newly initiated monitoring. If these same issues are replicated across the dozens of drugs simultaneously affected by shortages, the system-level stress can be large, absorbing precious clinician time and heightening risks of patient harm.

The clinical expertise and importance of pharmacists in navigating drug shortages are well-established, yet their capacity to act is often undercut by regulatory limitations. In many provinces, pharmacists are not permitted to substitute an equivalent medication without contacting the original prescriber, even when clinically appropriate options are available. These restrictions lead to frustrating delays and duplicate work for prescribers and patients. For example, in the case of the valsartan recall and shortage, pharmacists could have independently transitioned patients to medications such as candesartan, an agent in the same drug class. In appropriate situations, pharmacists could have even transitioned patients to an alternative hypertension medication entirely, such as ramipril. These substitutions are often well within the pharmacist’s training and judgment and should not require redundant authorization. Failing to support these interventions during a crisis amounts to a missed opportunity for efficient, patient-centred care and further weighs down an overstretched system.

## Compounding as a response to drug shortages

The 2022 shortage of pediatric analgesics provides a clear example of how pharmacists could have played a greater role through compounding. As children’s formulations of acetaminophen and ibuprofen disappeared from pharmacy shelves, families resorted to desperate and risky measures by splitting adult tablets at home or preparing their own suspensions.^4,5^ This improvisation led to a rise in poison control calls, highlighting the danger of leaving the public without professional guidance.^5^ Pharmacists, who possess both the knowledge and equipment to safely split doses or compound pediatric formulations, were well-positioned to respond. However, regulatory barriers prevented swift action. Without a new prescription, pharmacists in most jurisdictions could not legally prepare compounded alternatives, despite urgent clinical need.

Health Canada would, later that year, intervene and remove its objection to compounding over-the-counter pediatric analgesics without the need for a prescription.^6^ This impromptu response highlights a greater need for clarity and coordination between federal and provincial decision-makers around the ability to quickly leverage compounding as a response to drug shortages. In contrast, in the United States, pharmacists are automatically granted compounding authority during declared shortages.^7^ Canada’s lack of a similar mechanism leaves the system less responsive and increases the risk of patient harm.

Pharmacists across Canada routinely compound medications in both hospital and community settings. In hospitals, this may involve sterile intravenous preparations for patients with complex needs; in the community, pharmacists compound creams, suspensions, and other formulations when commercial products are inadequate. Despite these competencies, pharmacists are generally not permitted to initiate compounded alternatives during a shortage unless a physician first provides a new prescription. This restriction is particularly concerning given the time-sensitive nature of many shortage responses and the well-established safety record of pharmacists engaging in compounding. It reflects a legacy model of practice that fails to align authority with professional capability.

Another critical barrier to compounding is reimbursement. In Canada, compounded medications are often not reimbursed by public or private drug plans. Patients are frequently asked to pay out-of-pocket even when the compounded product is a direct substitute for a drug that is normally covered. This punishes patients for systemic failures beyond their control. It also disincentivizes pharmacists from providing these services, especially in low-income communities, resulting in an inequity of care. Reimbursement policies should be modernized to ensure that compounded medications for drugs that are temporarily unavailable are treated as medically necessary and financially covered.

## A broader crisis framework is necessary

Limitations to the pharmacist response to drug shortages are codified by national standards. The Canadian Association of Pharmacy Regulatory Authorities (CAPRA) directs provincial colleges to follow guidelines set by the National Association of Pharmacy Regulatory Authorities (NAPRA). While these standards are essential for ensuring quality and patient safety, they are insufficiently adaptable to emergency conditions. Canada lacks a “crisis” mechanism like other countries that could temporarily extend the pharmacist’s scope in defined and monitored circumstances. Importantly, most responses have looked to Health Canada to develop policies, but the ability to define the pharmacy scope of practice falls outside of their mandate. This type of mechanism could include risk-based criteria to permit therapeutic substitution or compounding without a new prescription, paired with documentation and physician notification to maintain collaborative care.

The fragmentation of regulatory frameworks across provinces adds further complexity (Table 1). While some jurisdictions, such as Ontario, permit pharmacists to alter dosage forms or strengths, they stop short of allowing therapeutic substitution. Alberta remains the only province where pharmacists can independently initiate prescriptions in response to a shortage.^8^ This inconsistency underlines the incoherence of Canada’s national drug strategy and creates variability in patient care.

**Table 1:** Pharmacist scope in Canada’s drug shortage response

	Adapt an existing prescription with a different dosage or formulation	Therapeutic substitution with a medication within the same therapeutic class*	Therapeutic substitution with a medication that is clinically equivalent regardless of therapeutic class	Independently initiate a prescription
British Columbia	✓	✓	✗	✗
Alberta	✓	✓	✓	✓
Saskatchewan	✓	✓	✗	✗
Manitoba	✓	✗	✗	✗
Ontario	✓	✗	✗	✗
Quebec	✓	✓	✗	✗
New Brunswick	✓	✓	✓	✗
Nova Scotia	✓	✓	✓	✗
Prince Edward Island	✓	✓	✓	✗
Newfoundland and Labrador	✓	✓	✗	✗
Yukon	✓	✓	✓	✗
Northwest Territories	✗	✗	✗	✗
Nunavut	✗	✗	✗	✗

* Therapeutic substitution is often defined as replacing the original drug with an alternative that is considered therapeutically equivalent. However, in some jurisdictions, therapeutic substitution is restricted to replacing the original drug with an alternative in the same drug class.

## Conclusion

The persistence of drug shortages in Canada and the growing expectation that they will become more frequent demand a fundamental shift in how pharmacists are integrated into the health system’s response. Pharmacists continue to be at the front-line in dealing with drug shortages and are uniquely positioned to play a central role in shortage mitigation, yet current regulations constrain their ability to act swiftly and effectively during times of crisis. A coordinated national framework is urgently needed to enable an expanded pharmacist scope during crises, harmonize interprovincial variation, and support the development of emergency protocols like those used in public health responses such as the COVID-19 pandemic. Drug shortages are no longer exceptional disruptions but a common occurrence. To meet this challenge, Canada must update its regulatory and financial frameworks to fully mobilize pharmacists to empower their ability to respond.

## References

[1] Health Canada. Drug shortages in Canada: fiscal year 2022 to 2023 in review. 2023. Available: https://www.canada.ca/en/health-canada/services/drugs-health-products/drug-products/drug-shortages/2022-2023-review.html (accessed May 21, 2025).

[2] Santhireswaran A Ho M Fuller K Gaudette E Burry L. Impact of supply chain disruptions and drug shortages on drug utilization: a scoping review protocol. PLoS One 2024;19:e0313298. doi:10.1371/journal.pone.0313298 PMC11530092 39485772

[3] Fenna J Chu C Hassan R Gomes T Tadrous M. Extent of a valsartan drug shortage and its effect on antihypertensive drug use in the Canadian population: a national cross-sectional study. CMAJ Open 2021;9(4):E1128-33. doi:10.9778/cmajo.20200232 PMC8673482 34876414

[4] Health Canada. Supply of acetaminophen, ibuprofen and cough and cold medicines: notice. 2023. Available: https://www.canada.ca/en/health-canada/services/drugs-health-products/drug-products/drug-shortages/information-consumers/supply-notices/acetaminophen-ibuprofen-cough-cold.html (accessed May 21, 2025).

[5] Zipursky JS Brown KA Khan S , et al. Pediatric dosing errors during a national shortage of fever and pain medications. N Engl J Med 2023;388(22):2099-2101. doi:10.1056/NEJMc2300561 37256982

[6] B.C. Ministry of Health. PharmaCare newsletter: November 2022 [PDF]. Government of British Columbia, November 1, 2022. Available: https://www2.gov.bc.ca/assets/gov/health/health-drug-coverage/pharmacare/newsletters/pharmacare_newsletter_november_2022.pdf (accessed May 21, 2025).

[7] U.S. Food and Drug Administration. Compounding when drugs are on FDA’s drug shortages list. 2024. Available: https://www.fda.gov/drugs/human-drug-compounding/compounding-when-drugs-are-fdas-drug-shortages-list (accessed May 21, 2025).

[8] Canadian Pharmacists Association. Scope of practice. 2025. Available: https://www.pharmacists.ca/advocacy/scope-of-practice/ (accessed May 21, 2025).

